# Fecal Nutrients Suggest Diets of Higher Fiber Levels in Free-Ranging than in Captive Proboscis Monkeys (*Nasalis larvatus*)

**DOI:** 10.3389/fvets.2017.00246

**Published:** 2018-01-19

**Authors:** Ikki Matsuda, Henry Bernard, Augustine Tuuga, Sen K. S. S. Nathan, John C. M. Sha, Ismon Osman, Rosa Sipangkui, Satoru Seino, Sanae Asano, Anna Wong, Michael Kreuzer, Diana A. Ramirez Saldivar, Marcus Clauss

**Affiliations:** ^1^Chubu University Academy of Emerging Sciences, Kasugai-shi, Japan; ^2^Wildlife Research Center, Kyoto University, Sakyo, Kyoto, Japan; ^3^Japan Monkey Centre, Inuyama, Japan; ^4^Institute for Tropical Biology and Conservation, Universiti Malaysia Sabah, Kota Kinabalu, Malaysia; ^5^Sabah Wildlife Department, Wisma Muis, Kota Kinabalu, Malaysia; ^6^School of Sociology and Anthropology Department, Sun Yat-sen University, Guang Zhou, China; ^7^Singapore Zoo, Wildlife Reserve Singapore, Singapore, Singapore; ^8^Zoorasia, Yohohama Zoological Gardens, Yokohama, Japan; ^9^Department of Animal Science and Resources, Nihon University, Kameino, Fujisawa, Japan; ^10^ETH Zurich, Institute of Agricultural Sciences, Zurich, Switzerland; ^11^Clinic for Zoo Animals, Exotic Pets and Wildlife, Vetsuisse Faculty, University of Zurich, Zurich, Switzerland

**Keywords:** colobine, fecal nutrient, captivity, folivore, foregut fermentation

## Abstract

Understanding the natural diet of species may provide useful information that can contribute to successful captive maintenance. A common problem experienced with captive foregut-fermenting primate (colobine) diets is that they are deficient in fiber and therefore highly digestible. This may contribute to gastrointestinal disorders often observed in zoos. An approach to obtain information relevant for the improvement of diets is to compare the nutrient composition of feces from free-ranging and captive individuals. In theory, fecal material can be considered a proxy for diet intake integrated over a certain period of time. We collected fecal samples from eight free-ranging proboscis monkey (*Nasalis larvatus*, a highly endangered colobine species) groups from a secondary forest along the Kinabatangan River and four from a mixed mangrove-riverine forest along the Garama River, Sabah, Borneo, Malaysia. We also collected fecal samples from 12 individual captive adult/sub-adult proboscis monkeys from three different zoos. We confirmed that feces from free-ranging monkeys contained more fiber and less metabolic fecal nitrogen than those from captive specimens, indicating a less digestible diet in the wild. Modifying the diets of captive colobines to include more fiber, comparable to those of free-ranging ones, may contribute to their health and survival.

## Introduction

Today, habitat destruction and poaching threaten nearly half of the world’s free-ranging primate species with extinction (1). Hence, conservation programs have become integral aspects of zoological management. An important issue in *ex situ* animal management is to determine the nutritional requirements of animals, to ensure that an appropriate diet is made available, and to facilitate their breeding (2). Foregut-fermenting primates, i.e., colobines, were historically difficult to maintain healthy in captivity, and they had shorter lifespans compared to free-ranging individuals (3–5). Free-ranging wild colobine monkeys are highly folivorous (6, 7); however, in captivity they have often been fed diets similar to those fed to frugivorous and/or omnivorous primates [e.g., Ref. (5, 8, 9)], which may lead to gastrointestinal disorders probably due to a less fibrous or too well-digestible diet (10–12), given that commercial fruits typically have high nutrient density compared to wild fruits (13–15).

Proboscis monkeys (*Nasalis larvatus*), endangered and endemic to Borneo, are the largest foregut-fermenting colobines. They consume leaves, fruits, and flowers in various proportions, although leaves generally dominate their diet [representing 38–92% of their diets: (16–20)]. Even compared to other colobines, these monkeys are notoriously difficult to maintain and breed in captivity, with the only notable successful long-term husbandry (1998–present) being at the Wildlife Reserves Singapore (21). Several other attempts to breed them have been made at zoos in non-tropical regions (5, 10, 22), and Yokohama Zoo, Japan, is the only non-tropical zoo that currently holds the species [2009–present: (23)].

To obtain information relevant for the improvement of diets of captive proboscis monkeys, one approach is to compare the nutrient composition of feces in free-ranging and captive individuals. To our knowledge, this approach has not yet been undertaken in primates, although in theory, it should be applicable to primates based on the accumulation of considerable knowledge regarding such nutrient analyses derived mostly from studies on grazing ruminant livestock (24). A notable exception is a study by Chapman et al. (25), that compared fecal nitrogen content (but not other nutrients) of free-ranging and captive colobines, suggesting that quantifying fecal nitrogen levels may be useful for assessing their habitat quality. However, their study did not differentiate fecal nitrogen derived from indigestible plant protein [neutral detergent fiber (NDF)-bound protein: (26)] and metabolic fecal nitrogen (MFN), a distinction of particular relevance in browsing animals (27). MFN consists predominantly of microbial nitrogen that is derived either from the degradation of plant protein or the incorporation of endogenous proteins (e.g., digestive enzyme residues) into microbial matter.

Here, we compared the fecal nutrient concentrations of free-ranging and captive proboscis monkeys and hypothesized that more fiber and lower levels of MFN would be found in the free-ranging specimens. A higher fiber content of the feces would result either from a higher proportion of fiber in the diet or from a lower digestibility/higher lignification of the fiber, both consistent with a lower energy density and digestibility of the diet. Without knowing the quantity and composition of food consumed and the quantity of feces defecated, fecal composition alone cannot be considered conclusive evidence for the composition and digestibility of a diet, because theoretically, different combinations of diet nutrient composition, intake, and digestibility can lead to the same fecal nutrient composition. Nevertheless, the use of fecal nitrogen [total fecal nitrogen (TFN)] as an indicator of diet quality has a long-standing tradition. A traditional view in herbivore ecology is that TFN can serve as a proxy for the protein content of the ingested diet. However, fecal protein levels represent, to a large proportion, microbial protein, and processes resulting in microbial growth do not directly reflect dietary protein levels, but rather overall diet digestibility (to which dietary protein levels are only one of many contributory factors); therefore, TFN should rather be considered as a proxy for the overall diet digestibility (28–30). In folivorous species that ingest a substantial amount of plant secondary compounds, TFN is compromised as an indicator by high proportions of indigestible N in the diet, and therefore, MFN is considered a better proxy (27).

## Materials and Methods

Fecal samples were collected from eight different free-ranging proboscis monkey groups between June and July 2015—four from monkeys inhabiting a secondary forest along the Kinabatangan River (118°30′E, 5°30′N) and four from monkeys present in a mixed mangrove-riverine forest along the Garama River (115°30′E, 5°21′N), Sabah, Malaysia. The samples were collected in the early morning (06:00–09:00 h) after the group left their sleeping trees (located the previous evening); all samples from within a group were pooled at the same point in time. Only fecal samples presumed to be (based on sample size) from adult individuals (31) were selected. Individual fecal samples were collected from 12 adult/sub-adult proboscis monkeys at three different zoos—four (male, one and females, three) from the Singapore Zoo (Singapore) in April 2014, four (males, three and female, one) from Lok Kawi Wildlife Park (Sabah, Malaysia) in July 2015 and four (males, two and females, two) from Yokohama Zoological Gardens Zoorasia (Yokohama, Japan) in September 2015. Several defecations of one individual were pooled until a sufficient sample volume for nutrient analysis was obtained (dry weight, ~15 g).

All fecal samples were sealed in plastic bags and stored at −20°C until oven-dried at 60°C for 60 h in the laboratory in state of Sabah, Malaysia, Singapore, and Japan, respectively. The dried samples were then milled and analyzed for total ash (TA), nitrogen/crude protein (CP), NDF, acid detergent fiber (ADF), acid detergent lignin (ADL), and acid-insoluble ash (AIA) using standard methods (32). Detergent fiber data are presented without residual ash. The MFN content of feces was calculated as TFN—undigested N from the diet quantified by analyzing the N content of the NDF fraction (NDF-N). Only TA, CP, and NDF were analyzed in Singapore Zoo samples.

Data were tested for normality (Kolmogorov–Smirnov test), followed by parametric *t*-tests for variables that were normally distributed, or nonparametric Mann–Whitney *U*-tests for variables that were not normally distributed, using SPSS (SPSS 23.0, IBM, Armonk, New York, NY, USA) to compare the fecal nutritional measurement between free-ranging and captive populations. Additionally, nonparametric correlations were tested using Spearman’s rank coefficient. The significance level was set at 0.05.

All research was conducted in compliance with guidelines for care and use of non-human primates by the Japan Monkey Centre and applicable Japan, Malaysian and Singaporean laws.

## Results

**Figure 1:**
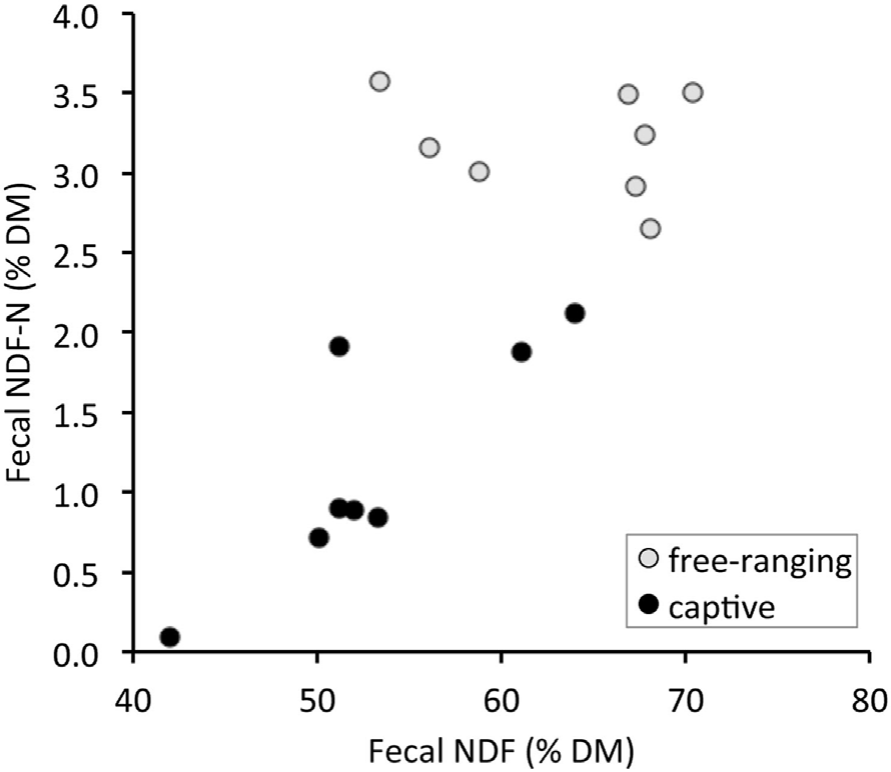
Relationship between total fecal nitrogen bound to neutral detergent fiber (NDF-N) and the concentration of neutral detergent fiber (NDF) in the feces of proboscis monkey groups (*Nasalis larvatus*) and captive proboscis monkey individuals.

There were significant differences in all fecal fiber, nitrogen, and TA contents between free-ranging and captive proboscis monkeys (Table 1). Generally, the levels of fiber, TFN, and NDF-N were higher in the feces of free-ranging animals. In contrast, MFN and TA were significantly higher in the feces of captive individuals. Although higher AIA levels were observed in the feces of captive individuals, differences were not significant; the SD for AIA was very high for captive individuals. When plotting fecal NDF-N against fecal NDF, there was a significant correlation in the data of captive monkeys (*R* = 0.71, *P* = 0.047, *n* = 8) but no correlation in the data of free-ranging specimens (*R* = −0.24, *P* = 0.570, *n* = 8) (Figure 1).

**Table 1:** Mean ± SD, median (25th, 75th percentiles) with number of observation of the contents of different constituents (% dry matter) in feces from free-ranging proboscis monkey groups (*Nasalis larvatus*) and captive proboscis monkey individuals.

	Free-ranging			Captive		

	N*	Mean ± SD	Median (25th, 75th percentiles)	*N*	mean ± SD	Median (25th, 75th percentiles)
NDF	8	63.6 ± 6.5	67.0 (56.7,68.0)^A^	12	51.8 ± 5.9	51.2 (48.3,53.0)^B^
ADF	8	43.0 ± 4.4^a^	43.0 (40.0,46.7)	8	36.9 ± 6.1^b^	37.9 (31.5,41.2)
ADL	8	35.2 ± 6.7	37.7 (31.8,39.4)^A^	8	25.1 ± 5.0	23.7 (23.0,25.3)^B^
TFN	8	4.3 ± 0.2^a^	4.3 (4.1,4.4)	12	3.7 ± 0.6^b^	3.9 (3.0,4.2)
NDF-N	8	3.2 ± 0.3^a^	3.2 (2.9,3.5)	8	1.2 ± 0.7^b^	0.9 (0.7,1.9)
MFN	8	1.1 ± 0.5	0.9 (0.7,1.6)^A^	8	2.4 ± 0.7	2.1 (2.0,2.4)^B^
TA	8	9.3 ± 2.0^a^	8.8 (7.6,10.6)	12	13.9 ± 4.1^b^	13.6 (10.4,15.8)
AIA	8	1.1 ± 1.1	0.7 (0.2,1.8)^A^	8	2.8 ± 4.0	1.2 (0.4,3.4)^B^

*All values in % dry matter*.

*For free-ranging animals, N denotes the number of different groups represented by a pooled fecal sample.

*NDF, neutral detergent fiber; ADF, acid detergent fiber; ADL, acid detergent lignin; TFN, total fecal nitrogen; NDF-N, nitrogen in NDF; MFN, metabolic fecal nitrogen; TA, total ash; AIA, acid-insoluble ash*.

*Within rows, superscripts refer to significant differences due to parametric *t*-test for normally distributed data (a, b) or due nonparametric *U*-test for not normally distributed data (A, B)*.

## Discussion

To determine an appropriate diet for captive animals, the nutrient composition of the diets of captive and free-ranging individuals is typically compared (14, 15, 33). However, this approach requires the sampling and analysis of a large number of food items in the wild, coupled with observations of the respective feeding frequency and quantity consumed to determine their overall dietary contribution (2). In contrast, fecal material represents an integrated sample over a certain period of diet intake, is easier to obtain, and requires fewer samples. In terms of nutritional information, a comparison of nutrient contents, particularly fiber, may be of more immediate relevance to the design of diets than alternative measurements like microbiome composition or hormone levels (34–37).

We confirmed the prediction that the feces from free-ranging monkey groups contained more fiber (higher NDF, ADF, ADL) and less MFN, suggesting a lower diet digestibility than those of captive individuals. Although in theory, different combinations of dietary fiber levels, amounts of food intake, and fecal excretion can lead to the same fecal nutrient concentrations, this theoretical range of possibilities is in reality confined by the fact that across a broad range of dietary fiber levels, higher fiber levels are typically associated with lower digestibility (38, 39), also in colobine monkeys (11). The results therefore indicate that free-ranging monkeys consume food items of lower digestibility than do captive monkeys. Although free-ranging proboscis monkeys carefully select leaves containing less fiber and more protein with higher *in vitro* digestibility (40–42), the nutritive quality of commercial fruits and vegetables fed to captive individuals is higher than that of the foods accessible to the free-ranging monkeys (14, 15, 33). Higher NDF levels in the feces of captive animals might be achieved by feeding more browse, concomitantly leading to higher NDF-N, as evident in Figure 1. In contrast, in free-ranging individuals whose diet exclusively comprises wild leaves and fruits, no comparable relationship between these two measures (TFN vs. NDF-N) was observed (Figure 1) because the diet items selected by free-ranging animals most likely varied in their NDF-N contents at concomitantly high NDF contents (26). This also supports the presence of differences in nutritional characteristics of the diets between free-ranging and captive individuals.

Previous studies have demonstrated that the production of well-shaped (healthy) solid feces in captive colobines requires an appropriate dietary intake of fiber (e.g., NDF) (10, 43). We suggest that modifying captive colobine diets so that the fiber intake is more similar to that of free-ranging individuals, may putatively enhance their health and survival in captivity. The captive proboscis monkeys in our study had fecal NDF contents (42–64% in dry matter) that were lower than those of free-ranging conspecifics (53–70%), but still far higher than those reported for other captive colobines—proboscis monkeys, 17% [mean of two different values: (22)]; Javan langurs (*Trachypithecus auratus*), 37% [mean of six different values: (10)], François langur (*T. françoisi*), 31% [mean of three different values: (43)] and 28–44%, Black-and-white colobus monkey (*Colobus guereza*), 28–51%, Northern douc langur (*Pygathris nemaeus*), 34–49% [based on experiments with a low-fiber and a high-fiber pelleted food: calculated from Ref. (44)]. This difference is most likely due to the feeding regime, which includes a higher proportion of browse than reported for other colobines (21). The experiments of Edwards and Ullrey (44) demonstrate that including high levels of fiber in the pelleted food compound is a factor that can contribute to achieve fecal fiber levels closer to free-ranging conditions than traditional, low-fiber primate pellets. Although differences in fecal fiber levels are likely to occur within a species, due to factors related to habitat, season, sex or reproductive status, the general magnitude of differences can serve as a convenient proxy of the appropriateness of any particular diet in captivity. Long-term feeding trials will be necessary to test whether more fibrous foods can truly reinforce the health and reproductive success in captive proboscis monkeys.

The diets of captive browsing ungulates are thought to contain higher AIA than those in the wild (45, 46). We observed a similar but not significant trend (given the large SD) in the feces of captive monkeys. However, because AIA is related to animal tooth wear affecting body condition, reproductive success and longevity in ungulates (47, 48), this trend might be worth considering for captive colobines, in general, for future studies. Because neither browse nor fruits and vegetables contain significant amounts of AIA, the most likely source of high AIA levels are compound feeds (45). Controlling AIA in such feeds may be a relevant future objective in the manufacturing of zoo foods for non-grazing species.

This study may shed light on the establishment of a constructive *in situ* and *ex situ* collaborative link to aid the management of dietary husbandry for captive colobines and possibly to provide necessary impetus for conservation and education initiatives, which will be beneficial for their long-term conservation. Further comparisons of fecal nutrient levels in other colobine species will be useful to establish targets for group- or species-specific fiber (and potentially other nutrients and minerals) supplementation.

## Conclusion

Lower fecal fiber contents in captive foregut-fermenting proboscis monkeys than those of free-ranging conspecifics were found, but they were still far higher than those reported in the literature for other captive foregut-fermenting primates. In addition, the feces of free-ranging proboscis monkey groups contained less MFN, indicating that the free-ranging proboscis monkeys consumed foods of lower digestibility compared to captive ones. To reduce the occurrence of gastrointestinal disorders and enhance health and survival, it may be recommendable to alter diets of captive animal diets to closer replicate fecal fiber levels found in free-ranging specimens.

## Author Contributions

IM and MC conceptualized the initial idea; IM, HB, IO, and SS performed the fecal sampling; AT, SKSSN, JCMS, RS, AW, and DARS arranged the sampling in the wild/zoo; IM, SA, and MK performed the nutritional analyses, IM and MC performed and interpreted the statistical analysis; IM, MC, and MK drafted the manuscript; and IM organized the projects. All authors contributed to the final version of the manuscript.

## Conflict of Interest Statement

The authors declared no potential conflicts of interest with respect to the research, authorship, and/or publication of this article.
